# Arsenic trioxide-induced apoptosis contributes to suppression of viral reservoir in SIV-infected rhesus macaques

**DOI:** 10.1128/spectrum.00525-23

**Published:** 2023-09-11

**Authors:** Yizi He, Chunxiu Wu, Zijian Liu, Yudi Zhang, Fengling Feng, Zihan Lin, Congcong Wang, Qing Yang, Ziyu Wen, Yichu Liu, Fan Zhang, Yanqin Lin, Hao Zhang, Linbing Qu, Linghua Li, Weiping Cai, Caijun Sun, Ling Chen, Pingchao Li

**Affiliations:** 1 Guangdong Laboratory of Computational Biomedicine, Center for Infection and Immunity, Guangzhou Institutes of Biomedicine and Health, Chinese Academy of Sciences, Guangzhou, China; 2 University of Chinese Academy of Sciences, Beijing, China; 3 School of Public Health (Shenzhen), Shenzhen Campus of Sun Yat-sen University, Shenzhen, China; 4 Guangzhou Eighth People’s Hospital, Guangzhou Medical University, Guangzhou, China; National Institutes of Health, Bethsda, Maryland, USA

**Keywords:** arsenic trioxide, HIV/SIV, latent viral reservoir, rhesus macaques, apoptosis

## Abstract

Latent viral reservoir is recognized as the major obstacle to achieving a functional cure for HIV infection. We previously reported that arsenic trioxide (As_2_O_3_) combined with antiretroviral therapy (ART) can reactivate the viral reservoir and delay viral rebound after ART interruption in chronically simian immunodeficiency virus (SIV)-infected macaques. In this study, we further investigated the effect of As_2_O_3_ independent of ART in chronically SIV-infected macaques. We found that As_2_O_3_-only treatment significantly increased the CD4/CD8 ratio, improved SIV-specific T cell responses, and reactivated viral latency in chronically SIVmac239-infected macaques. RNA-sequencing analysis revealed that As_2_O_3_ treatment downregulated the expression levels of genes related to HIV entry and infection, while the expression levels of genes related to transcription initiation, cell apoptosis, and host restriction factors were significantly upregulated. Importantly, we found that As_2_O_3_ treatment specifically induced apoptosis of SIV-infected CD4^+^ T cells. These findings revealed that As_2_O_3_ might not only impact viral latency, but also induce the apoptosis of HIV-infected cells and thus block the secondary infection of bystanders. Moreover, we investigated the therapeutic potential of this regimen in acutely SIVmac239-infected macaques and found that As_2_O_3_ + ART treatment effectively restored the CD4^+^ T cell count, delayed disease progression, and improved survival in acutely SIV-infected macaques. In sum, this work provides new insights to develop As_2_O_3_ as a component of the “shock-and-kill” strategy toward HIV functional cure.

**IMPORTANCE:**

Although antiretroviral therapy (ART) can effectively suppress the viral load of AIDS patients, it cannot functionally cure HIV infection due to the existence of HIV reservoir. Strategies toward HIV functional cure are still highly anticipated to ultimately end the pandemic of AIDS. Herein, we investigated the direct role of As_2_O_3_ independent of ART in chronically SIV-infected macaques and explored the underlying mechanisms of the potential of As_2_O_3_ in the treatment of HIV/SIV infection. Meanwhile, we investigated the therapeutic effects of ART+As_2_O_3_ in acutely SIVmac239-infected macaques. This study showed that As_2_O_3_ has the potential to be launched into the “shock-and-kill” strategy to suppress HIV/SIV reservoir due to its latency-reversing and apoptosis-inducing properties.

## INTRODUCTION

It has been more than 40 years since the first case report of an AIDS patient (1); however, we are still far from achieving a functional cure for human immunodeficiency virus (HIV) (2, 3). Currently, the level of plasma HIV RNA can be effectively controlled under detecting limitations with diverse antiretroviral therapy (ART) drugs targeting the separate processes of the viral life cycle (4, 5). Clinically, it is worth noting that long-acting combination drugs such as Cabenuva have been launched to ameliorate the plight of patients (6). Nevertheless, strategies for HIV functional cure are still highly anticipated to ultimately end the pandemic of AIDS.

“Shock and kill” is known as initiating the transcription of the integrated viral genome through latency-reversing agents (LRAs) and eradicating the reactivated virus with follow-up killing strategies (7). Currently, a variety of LRAs are under investigation, including Toll-like receptor (TLR) agonists, histone deacetylase inhibitors, DNA methyltransferase inhibitors, histone methyltransferase inhibitors, bromodomain inhibitors, PI3K/Akt pathway inhibitors, and protein kinase C agonists (8 – 10). However, there is no optimal LRA that can be applied clinically due to inefficient latency reversal *in vivo*, potential cytotoxicity, and extensive T cell activation (7, 11). In previous studies, we and other groups found that As_2_O_3_ significantly reactivated HIV/simian immunodeficiency virus (HIV/SIV) latency without causing inflammation and T cell activation (12, 13). We also observed that ART+As_2_O_3_ treatment restored CD4^+^ T cell counts, improved SIVmac239-specific T cell immune responses, and prolonged the time of viral load rebound in chronically SIVmac239-infected rhesus macaques after ART interruption. Half of the macaques in the ART+As_2_O_3_ group achieved an eighty-day-long remission of viremia. More importantly, the expression of CCR5 on CD4^+^ T cells was downregulated after As_2_O_3_ treatment, which implied a lower vulnerability of host cells to HIV/SIV infection (12). However, the effect of As_2_O_3_ independent of ART *in vivo* is still unclear, as is the mechanism behind the therapeutic potential of As_2_O_3_.

With the development of detection technology and the intensive monitoring of susceptible populations, patients infected by HIV can be diagnosed much earlier. What we can learn from the case of Mississippi infant is that even ART started as early as 30 h after exposure failed to achieve a cure. Nevertheless, it demonstrated the significance of initiating treatment during the acute infection stage (14). Benefits such as preserving the immune response (15), improving B cell responses (16), and facilitating the decay of the HIV reservoir (17, 18) have been shown in a few studies when ART was initiated during the early stage of HIV infection. It is reasonable to anticipate that the combination of ART with multiple agents during acute infection has the potential to achieve HIV remission. Whether ART+As_2_O_3_ treatment during the acute stage of infection shrinks the latent HIV reservoir remains unknown.

In this study, we investigated the effects of As_2_O_3_-only treatment in chronically SIVmac239-infected macaques. Moreover, we conducted RNA sequencing and *in vitro* experiments using CD4^+^ T cells from SIVmac239-infected macaques and HIV latency cell lines pretreated with As_2_O_3_ to explore the underlying mechanisms. We also investigated the effects of ART+As_2_O_3_ treatment in acutely SIVmac239-infected macaques to determine the advantages of launching As_2_O_3_ in acute HIV/SIV infection.

## RESULTS

### As_2_O_3_-only treatment elevated the CD4/CD8 ratio and SIVmac239-specific immune responses in chronically infected rhesus macaques

**Fig 1:**
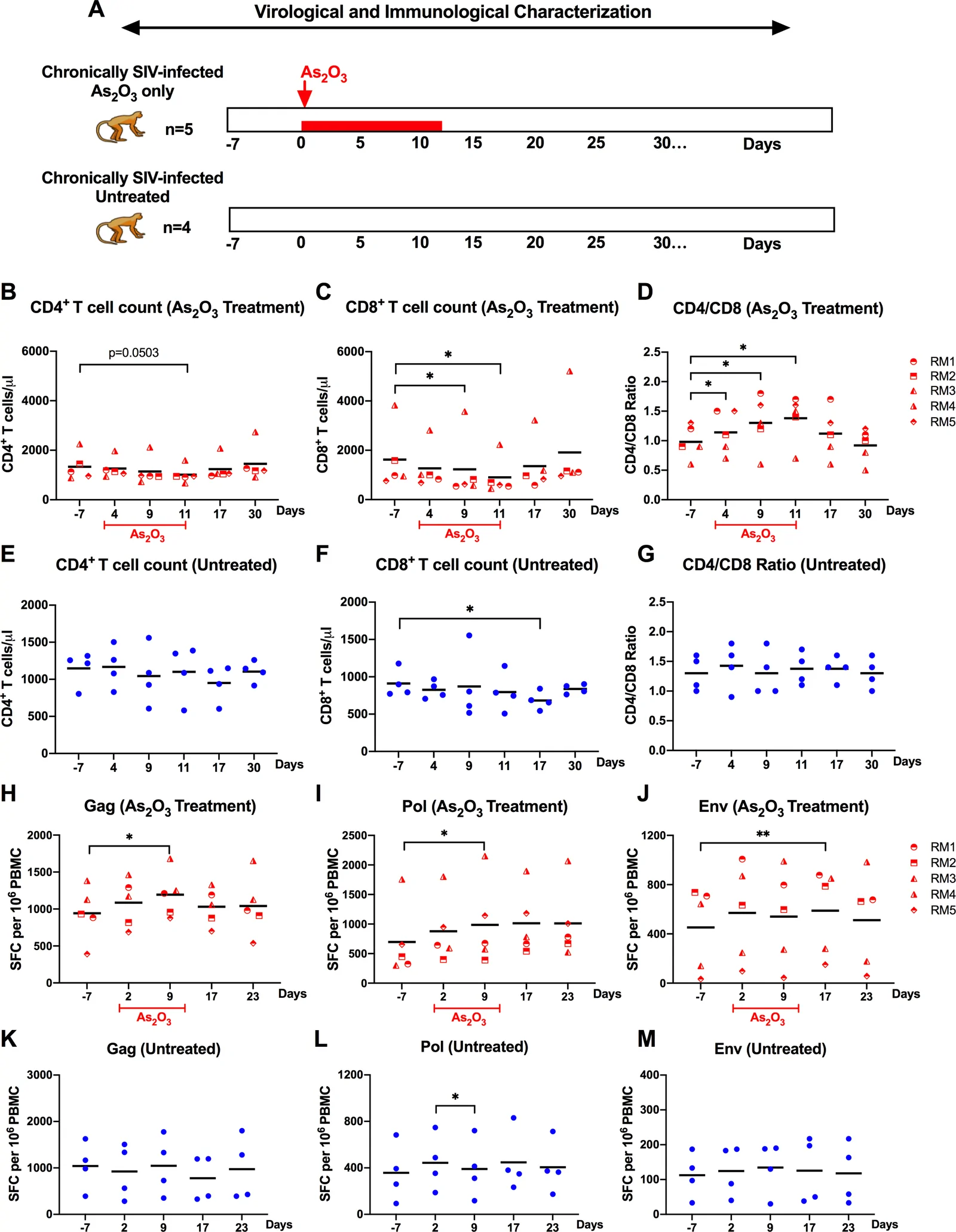
Dynamics of T cell counts and SIVmac239-specific immune responses after As_2_O_3_-only treatment in chronically SIVmac239-infected rhesus macaques. (**A**) Experimental design during the As_2_O_3_-only treatment phase. Five chronically SIV-infected macaques were treated with As_2_O_3_ alone for 12 days without ART, while four chronically SIV-infected macaques were untreated as controls. As_2_O_3_ (0.16 mg/kg) was administered by intravenous drip daily. Blood samples and peripheral blood mononuclear cells (PBMCs) were collected and subjected to virological and immunological analysis throughout the study. (**B–D**) The number of CD4^+^ T cells and CD8^+^ T cells and the CD4/CD8 ratio of the macaques in the As_2_O_3_-only group were monitored by flow cytometry at −7, 4, 9, 11, 17, and 30 days post treatment. (**E–G**) Dynamics of CD4^+^ and CD8^+^ T cell counts, and the CD4/CD8 ratio in the untreated group at the same time points. (**H–J**) PBMCs were isolated by density gradient centrifugation with Lymphoprep and then stimulated with SIVmac239 Gag, Pol, and Env peptides. The specific IFN-γ-secreted spot-forming cells (SFCs) per million PBMCs at different time points in the As_2_O_3_-only group were measured by ELISpot assay. (K–M) SIVmac239 Gag-, Pol-, and Env-specific IFN-γ-secreted spot-forming cells (SFCs) per million PBMCs at different time points in the untreated group. All data are presented as the grand mean. *P < 0.05, **P < 0.01.

**Fig 2:**
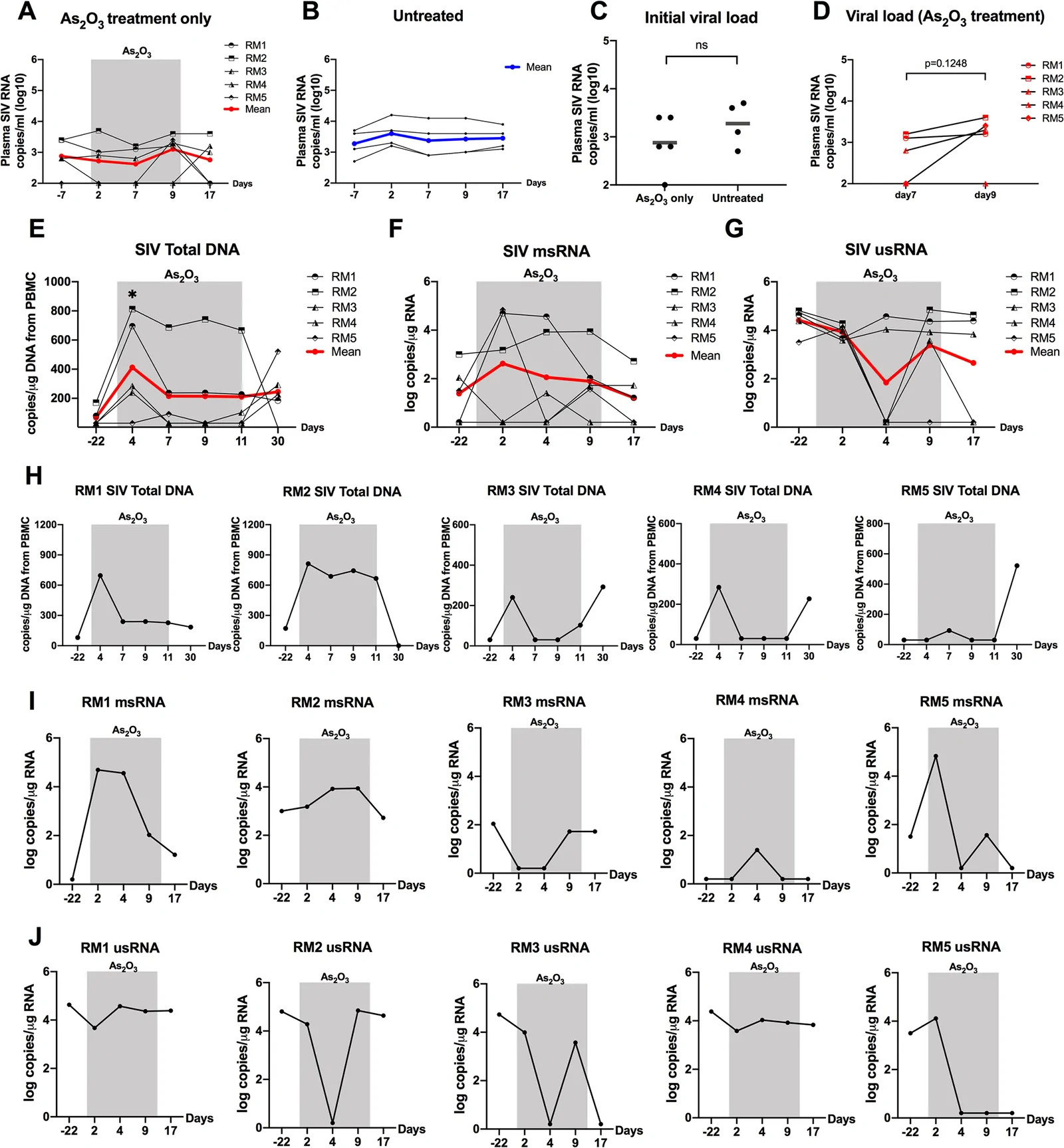
As_2_O_3_-only treatment-induced SIV expression in chronically SIVmac239-infected rhesus macaques. (**A, B**) The plasma viral load (log SIV RNA copies per mL plasma) of the As_2_O_3_-only group and the untreated group during the study period was monitored through quantitative PCR assay. The red line and the blue line represent the mean value of all macaques in each group. The gray shading represents the period of As_2_O_3_ administration. (**C**) Comparisons of the initial level of the viral load between the As_2_O_3_-only group and the untreated group. (**D**) Comparisons of the viral load in the As_2_O_3_-only group between day 7 and day 9 post treatment. (**E, H**) Genomic DNA was extracted from macaques PBMCs. The levels of SIV gag total DNA at different time points in the As_2_O_3_ group were detected by quantitative PCR assay. The gray shading represents the period of As_2_O_3_ administration. The red line represents the mean value of all macaques in the As_2_O_3_-only group. (**F, G, I, J**) RNA from PBMCs was separated and then reverse-transcribed. The levels of multiple spliced SIV RNA (**F, I**) and unspliced SIV RNA (**G, J**) at different time points during As_2_O_3_ treatment were detected through quantitative PCR assay. The gray shading represents the period of As_2_O_3_ administration. The red line represents the mean value of all macaques in the As_2_O_3_-only group. *P < 0.05.

In our previous study, we demonstrated the effects of ART+As_2_O_3_ combination therapy in chronically SIVmac239-infected rhesus macaques (12). To investigate the direct effect of As_2_O_3_ on HIV/SIV *in vivo*, nine Chinese rhesus macaques with chronic SIVmac239 infection for more than 2 years were used, and the plasma viral loads of these macaques were no more than 4 log10 and reached stable setpoints. Five of these macaques were treated with As_2_O_3_ alone for 12 days without ART, while four of them were untreated as controls (Fig. 1A). There was no significant difference in initial viral load between the As_2_O_3_-only group and the untreated group (Fig. 2C). One macaque (RM 4) with a significantly lower plasma viral load (≤2 log10) was assigned to the As_2_O_3_-only group to determine whether As_2_O_3_ treatment can reactivate the viral reservoir of individuals who control the virus well spontaneously. Blood samples of As_2_O_3_-only treated macaques were collected at different time points for immunological and virological detection (Fig. 1A). We first assessed whether As_2_O_3_-only treatment affects the immune response in chronically SIVmac239-infected macaques. To monitor the dynamics of T cell counts, we measured the quantities of different T cell subsets through flow cytometry at multiple time points. The results showed that the CD4^+^ T cell counts of rhesus macaques in the As_2_O_3_-only group showed a downward trend during the course of As_2_O_3_ administration (Fig. 1B), while the CD8^+^ T cell counts decreased significantly during treatment (Fig. 1C), but both recovered after the termination of treatment (Fig. 1B and C). It was reported that the CD4/CD8 ratio was a valid predictor of immune reconstitution prognosis (19). Although the number of T cells had a decreasing trend during As_2_O_3_ treatment, the ratio of CD4/CD8 was significantly increased (Fig. 1D), which proved that the function of the immune system was relatively stable during As_2_O_3_ treatment. By comparison, the T cell counts and CD4/CD8 ratio in untreated SIV-infected macaques showed no significant changes between day 7 and day 11 (Fig. 1E through G).

Moreover, we investigated SIVmac239-specific immunity through an IFN-γ-mediated ELISpot assay and found that the SIVmac239 Gag-, Pol-, and Env-specific spot-forming cells (SFCs) of macaques increased significantly after As_2_O_3_-only treatment (Fig. 1H through J), while no significant elevation was observed in the untreated macaques (Fig. 1K through M). This suggested that As_2_O_3_ treatment alone can effectively improve the SIV-specific immunity of macaques and promote immune reconstruction.

### As_2_O_3_-only treatment induced reactivation of the SIV latent reservoir in chronically SIVmac239-infected rhesus macaques

We next evaluated virological factors such as SIV viral load in plasma, SIV total Gag DNA, cell-associated multiple spliced SIV RNA (msRNA), and cell-associated unspliced SIV RNA (usRNA). The change in viral load was not significant; however, four macaques (except RM4) in the As_2_O_3_-only group had an increased viral load on day 9 compared to day 7 (Fig. 2A through D). During the same period, a plasma viral blip was observed in one macaque (RM5) in the As_2_O_3_-only group (Fig. 2D). To monitor the dynamics of the SIV reservoir during As_2_O_3_ treatment, we performed real-time fluorescent quantitative PCR and nested PCR to detect the changes in SIV total Gag DNA and cell-associated RNA in peripheral blood mononuclear cells (PBMCs) at different time points. The mean level of SIV total Gag DNA showed an increasing trend during the As_2_O_3_ treatment (Fig. 2E). All macaques treated with As_2_O_3_ showed upregulation of SIV total Gag DNA during treatment (Fig. 2H). Moreover, the level of SIV msRNA during the treatment process was also elevated in four out of five macaques, which means that As_2_O_3_ treatment promoted the production of early viral transcription products (Fig. 2F and I). At the same time, SIV usRNA, which reflects the formation of late viral transcripts, also showed a transient upward trend at different time points during As_2_O_3_ treatment (Fig. 2G and J). However, the mean level of SIV usRNA presented a decreasing trend (Fig. 2G). After the withdrawal of As_2_O_3_, the SIV usRNA level of five rhesus macaques returned to the initial level or even lower (Fig. 2J). The results demonstrated that As_2_O_3_ alone can reactivate the SIV latent reservoir in chronically SIVmac239-infected macaques during the treatment phase.

### As_2_O_3_ treatment regulated the expression of genes related to HIV entry, transcription initiation, cell apoptosis, and host restriction factors

**Fig 3:**
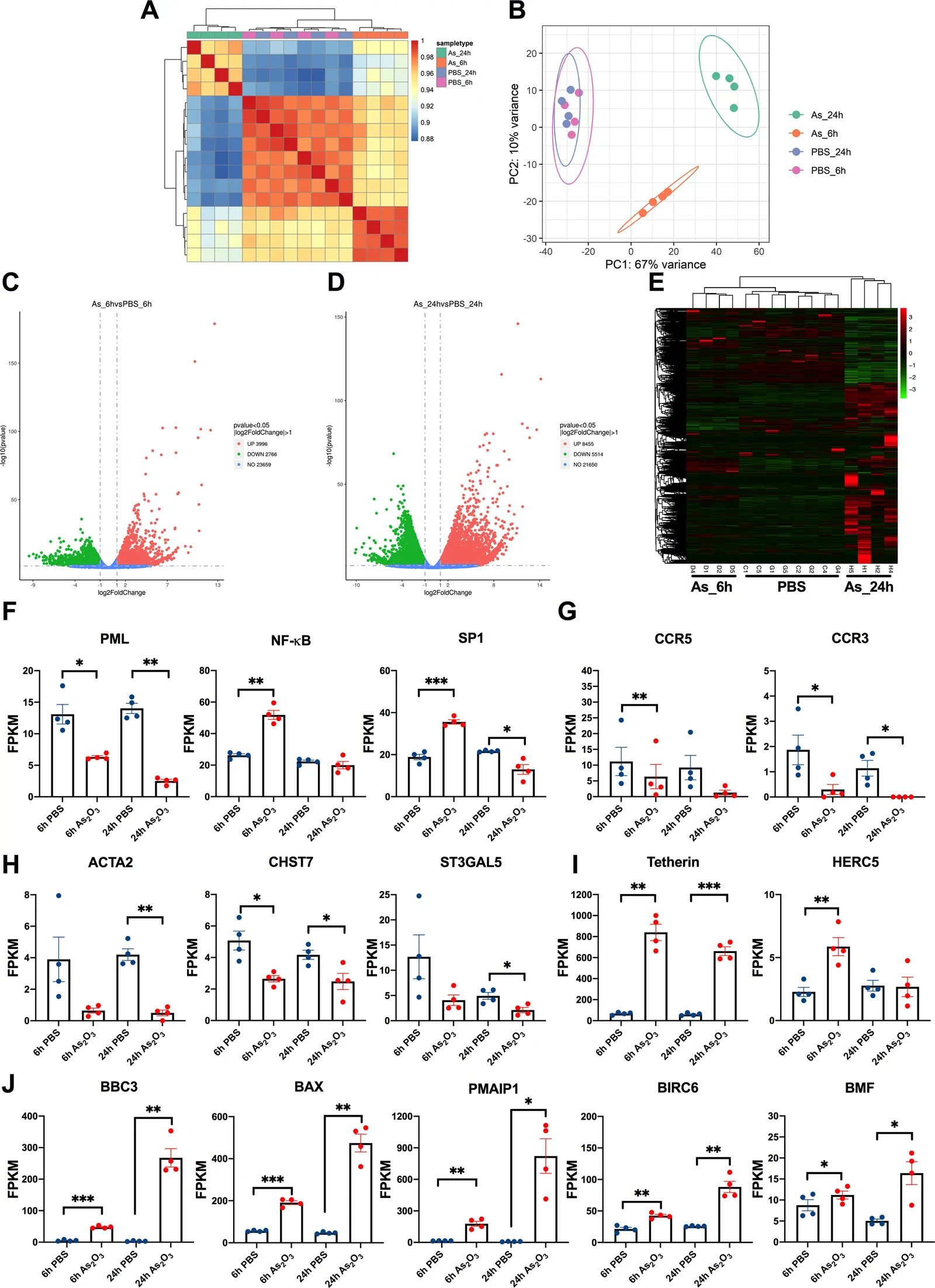
Transcriptomics of As_2_O_3_-treated primary CD4^+^ T cells from SIVmac239-infected rhesus macaques. CD4^+^ T cells from SIVmac239-infected macaques were sorted to perform RNA sequencing. These cells were treated with As_2_O_3_ (12.5 µM) or PBS for 6 h and 24 h before analysis. (**A**) The Pearson correlations between the 16 samples in As_6 h (*n* = 4), As_24 h (*n* = 4), PBS_6 h (*n* = 4), and PBS_6 h (*n* = 4) were confirmed. The correlation of gene expression levels between samples was an important indicator to test the reliability of experiments and to test whether the selection of samples was reasonable. The closer the correlation value was to 1, the higher the similarity of expression patterns between samples was. (**B**) Results of principal component analysis (PCA) according to the fragments per kilobase of exon model per million mapped fragments (FPKM). PCA is commonly used to assess the differences between groups and the relations within one group. (**C, D**) Analysis of differentially expressed genes (DEGs) in the As_2_O_3_ groups and PBS groups at 6 h (**C**) and 24 h (**D**) was performed using the DESeq2 R package (1.20.0). Benjamini and Hochberg’s approach was used to adjust the *P-*values for controlling the false discovery rate. Genes with a *P*-value < 0.05 were considered differentially expressed. (**E**) Hierarchical clustering and heat map of the DEGs between the As_2_O_3_ groups and the PBS groups. The abscissa is the sample name, while the ordinate is the normalized value of FPKM. The redder the color, the higher the expression level, and the greener, the lower the expression level. (**F–H**) Expression levels of genes that were associated with the initiation of HIV/SIV transcription (**F**), HIV/SIV entry (**G**), and promotion of HIV/SIV infection (**H**). (**I**) Expression level of host restriction factor genes. (**J**) Expression level of genes related to apoptosis promotion. All data are presented as the mean ± SD (*P < 0.05, **P < 0.01, ***P < 0.001). The expression level of genes was represented by FPKM.

As we have explored the potential effects of As_2_O_3_ treatment in chronically SIVmac239-infected rhesus macaques, we found that As_2_O_3_ treatment not only plays a role in HIV/SIV transcription but also enhances the immunity of macaques. These results inspired us to further explore the underlying mechanisms through bioinformatic technologies. CD4^+^ T cells from four SIVmac239-infected rhesus macaques were isolated by CD4 microbeads and treated with As_2_O_3_ (12.5 µM) for 6 h and 24 h *in vitro,* respectively. Then, the samples were collected to conduct RNA-seq analysis. The Pearson correlation of the samples in the same group was confirmed (Fig. 3A), while the principal component analysis (PCA) also showed a relevant correlation between the samples (Fig. 3B). By comparison with the PBS groups, As_2_O_3_-treated CD4^+^ T exhibited 6,762 differentially expressed genes (DEGs) at 6 h and 13,969 DEGs at 24 h (Fig. 3C and D). Samples treated with As_2_O_3_ were clustered and showed a distinct expression profile from samples treated with PBS (Fig. 3E). We also performed enrichment analysis based on DEGs. The top 30 terms in gene ontology (GO) analysis and the top 20 enriched pathways in the Kyoto Encyclopedia of Genes and Genomes (KEGG) database are listed in this study (Fig. S1). We found that DEGs were enriched in biological processes such as antigen processing and presentation, immune response, and pathways such as p53 signaling after treatment with As_2_O_3_ for 6 h (Fig. S1A and C).

Consistent with previous studies, genes such as PML (20), NF-κb (13), and SP1 (21), which are related to promoting the transcription of HIV/SIV latency, changed significantly after As_2_O_3_ treatment (Fig. 3F). Moreover, the CCR5 receptor (22) and CCR3 receptor (23), which play essential roles during HIV/SIV entry, were downregulated after As_2_O_3_ treatment at 6 h or at both time points (Fig. 3G), which is similar to our *in vivo* results when chronically SIVmac239-infected rhesus macaques were treated with ART+As_2_O_3_ (12). The expression levels of ACTA2, ST3GAL5, and CHST7 were also significantly downregulated after treatment with As_2_O_3_ (Fig. 3H). These genes were all previously reported to promote HIV infection (24). Host restriction factors were reported to be the first line of defense against viral pathogens. We found that As_2_O_3_ administration increased the expression levels of tetherin (BST2) and HERC5 (Fig. 3I). Tetherin can prevent virus release by tethering newly budding virions to the plasma membrane (25, 26), while HERC5 can inhibit the assembly of HIV by ISGylation of the Gag protein (27).

In addition to the genes related to the viral life cycle, genes associated with apoptosis differed greatly between the PBS groups and As_2_O_3_ treatment groups. Proapoptosis genes such as BBC3, Bax, PMAIP1, BIRC6, and BMF were all upregulated in the presence of As_2_O_3_ (Fig. 3J). BBC3, Bax, and PMAIP1 all belong to the p53-signaling pathway, which is one of the major apoptosis signaling pathways (28).

To verify the accuracy of our findings, we performed quantitative PCR on cDNA samples from Jurkat and Jurkat-Lat HIV-1 full-length clone A10.6 cell lines pretreated with medium, As_2_O_3_ (12.5 µM), and P + I (phorbol myristate acetate 40 ng/mL + ionomycin 1,000 ng/mL) for 24 h. The results showed that As_2_O_3_ treatment upregulated the expression of tetherin, NF-κB, and BBC3, and downregulated the expression of PML, CCR5, and SP1 (Fig. S2). These findings are consistent with the results of RNAseq experiments.

Overall, As_2_O_3_ treatment not only affects the transcription of the HIV/SIV reservoir but also decreases the expression of viral receptors on CD4^+^ T cells and genes related to promoting HIV infection. Moreover, antiviral host restriction factors were also upregulated to help defend against infection. The elevated level of apoptotic effects on SIV-infected CD4^+^ T cells after As_2_O_3_ treatment may also help to eliminate HIV/SIV-infected cells.

### As_2_O_3_ treatment specifically induced apoptosis of the CD4^+^ T cells from SIV-infected macaques and the HIV latency T cell lines

We found that differentially expressed genes were enriched in pathways related to apoptosis after As_2_O_3_ treatment. We next investigated whether As_2_O_3_ treatment could specifically induce apoptosis of latent reservoir cells. In this study, for primary cells from macaques, we used propidium iodide (PI) and FITC-conjugated Annexin V to distinguish between early apoptosis and late apoptosis. Early apoptotic cells were defined as Annexin V-positive and PI-negative, while late apoptotic cells were defined as Annexin V/PI-double-positive. For cell lines such as Jurkat-Lat HIV-1 full-length clone A10.6, which contains GFP signals, we used 7-amino-actinomycin (7-AAD) and PE-conjugated Annexin V to analyze early and late apoptotic cells. Early apoptotic cells were defined as Annexin V-positive and 7-AAD-negative, while late apoptotic cells were defined as Annexin V/7-AAD-double-positive.

**Fig 4:**
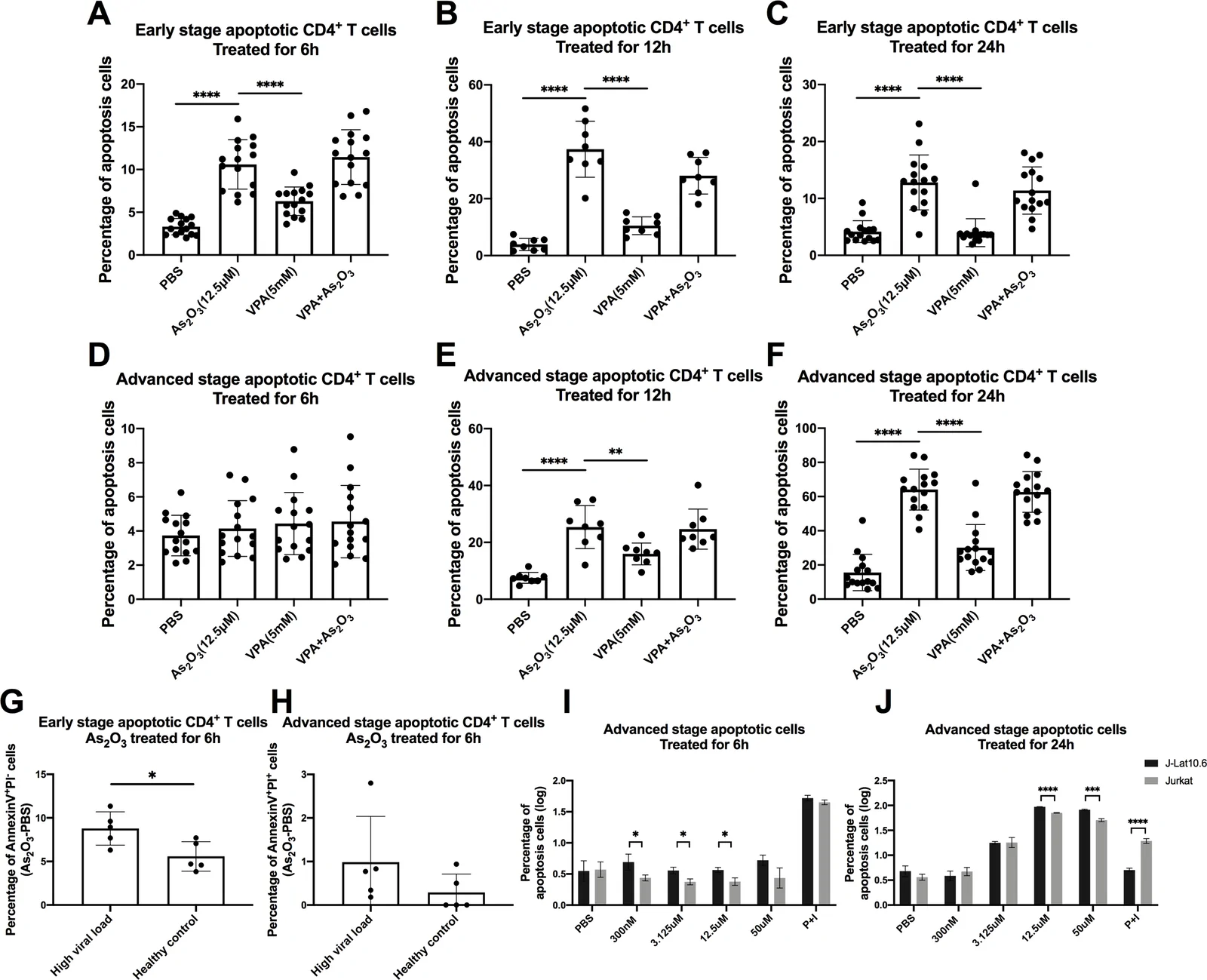
As_2_O_3_treatment *in vitro* induced a higher percentage of apoptosis toward CD4^+^ T cells from rhesus macaques with severe viremia. (**A-F**) Primary CD4^+^ T cells from SIVmac239-infected macaques and healthy macaques were separated by magnetic-activated cell separation. Samples from each macaque were all treated with PBS, As_2_O_3_ (12.5 µM), valproic acid (VPA, 5 mM), and As_2_O_3_ (12.5 µM) +VPA (5 mM). Apoptotic cells were monitored using a FITC/PE Annexin V Apoptosis Detection Kit I via flow cytometry. (**A–C**) Comparisons of the percentage of early stage apoptotic cells between groups with different treatments for 6 h (**A**), 12 h (**B**), and 24 h (**C**). (**D–F**) Comparisons of the percentage of advanced stage apoptotic cells between groups with different treatments for 6 h (**D**), 12 h (**E**), and 24 h (**F**). Early apoptotic cells were labeled Annexin V positive and propidium iodide (PI) negative, while advanced apoptotic cells were Annexin V positive and PI positive. (**G, H**) The percentage of early (**G**) and advanced (**H**) apoptotic CD4^+^ T cells after As_2_O_3_ treatment for 6 h among two groups of macaques with different levels of viremia. Enrolled individuals included five macaques with a viral load >5.6 log and five healthy uninfected macaques. To eliminate the intrinsic influences, the final values used here for each macaque were the percentage of apoptosis after As_2_O_3_ treatment minus that after PBS treatment. (**I, J**) Jurkat-Lat HIV-1 full-length clone A10.6 cells (J-Lat10.6) and Jurkat T cells were treated with PBS, As_2_O_3_ (300 nM, 3.125 µM, 12.5 µM, 50 µM), and P + I (phorbol myristate acetate 40 ng/mL + ionomycin 1000 ng/mL) for 6 h and 24 h, respectively. Then, the percentage of apoptotic cells was monitored by flow cytometry. 7-Amino-actinomycin (7-AAD)-positive and annexin V-positive cells were regarded as advanced-stage apoptotic cells in this study. For Fig. 4I and J, each experiment was performed in triplicate and repeated three times. All data are presented as the mean ± SD (*P < 0.05, **P < 0.01, ***P < 0.001, ****P < 0.0001).

Primary CD4^+^ T cells from SIV-infected macaques were used to evaluate the apoptosis-inducing efficacy of As_2_O_3_. Purified CD4^+^ T cells were treated with PBS, As_2_O_3_ (12.5 µM), valproic acid (VPA, 5 mM), and As_2_O_3_ (12.5 µM) +VPA (5 mM) *in vitro* for 6 h, 12 h, and 24 h. The results showed that As_2_O_3_ treatment significantly induced the early apoptosis of CD4^+^ T cells from SIV-infected macaques and healthy macaques at all three time points (Fig. 4A through C) and significantly induced the advanced stage of apoptosis at 12 h and 24 h (Fig. 4E and F). The phenomenon of inducing advanced apoptosis of CD4^+^ T cells appeared to occur in a time-dependent manner (Fig. 4D through F). When combined with VPA, the proapoptotic efficiency was comparable to that of As_2_O_3_ treatment alone.

We next analyzed whether the proapoptotic efficiency of As_2_O_3_ treatment differs between the two groups based on the level of viremia. CD4^+^ T cells were collected from either healthy macaques (*n* = 5) or SIVmac239-infected macaques with viral loads higher than 10^6^ copies/mL (*n* = 5). To our surprise, we found that CD4^+^ T cells from macaques with a high SIV viral load showed a significantly higher level of early apoptosis than those from healthy donors at 6 h (Fig. 4G), while the advanced stage of apoptosis also showed a similar viral load-dependent manner (Fig. 4H). These results indicated that As_2_O_3_ treatment may tend to eliminate cells from individuals with a high viral load, which is positively correlated with latent reservoir size.

The Jurkat-Lat T cell line is a commonly used model to mimic HIV-1 latency. Different concentrations of As_2_O_3_ were used to treat Jurkat-Lat HIV-1 full-length clone A10.6 cells (J-Lat10.6) and Jurkat cells. We found that As_2_O_3_ treatment induced a significantly higher level of apoptosis in J-Lat10.6 cells than in Jurkat cells at the same concentration (Fig. 4I and J).

In conclusion, As_2_O_3_ treatment promotes the apoptosis of primary CD4^+^ T cells from SIV-infected macaques and HIV latency T cell lines, while this effect shows a preference for cells containing latent reservoirs. This might broaden the use of As_2_O_3_ treatment in HIV/SIV infection.

### Delayed progression of viremia by ART+As_2_O_3_ therapy in acutely SIV-infected rhesus macaques after ART interruption

**Fig 5:**
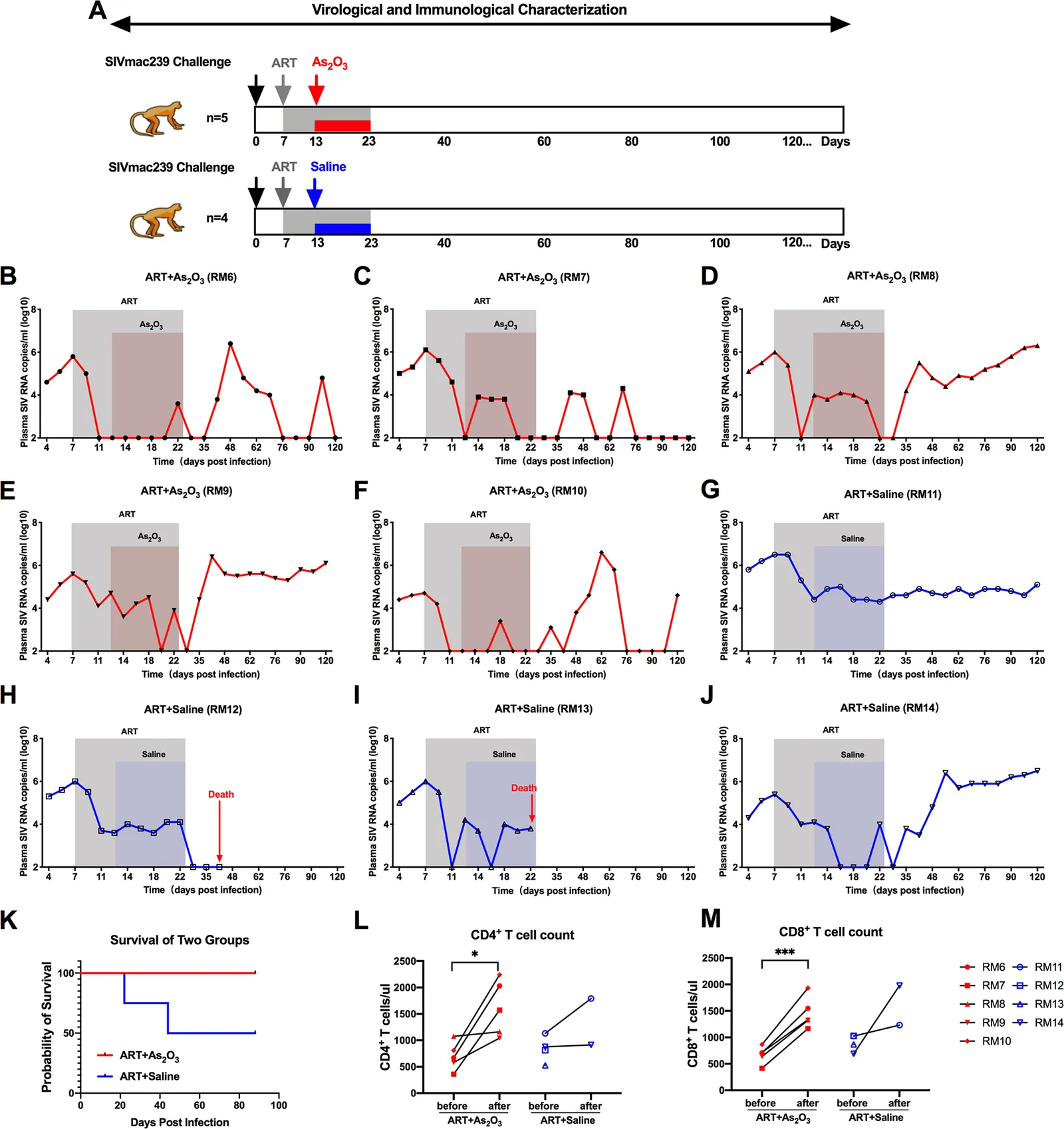
Delayed progression of viremia by ART +As_2_O_3_ therapy in acutely SIVmac239-infected rhesus macaques after ART interruption. (**A**) Experimental design during the ART2Saline and ART+As_2_O_3_ treatment phases. Nine rhesus macaques were infected by SIVmac239 intravenously on day 0. Five macaques were assigned to the ART+As_2_O_3_ group, while the other four macaques were assigned to the ART +saline group. ART was initiated for all of the macaques on day 7 and was withdrawn on day 23. As_2_O_3_ treatment started on day 13 and stopped on day 23. (**B–F**) Viral load (plasma SIV RNA) levels of the five macaques in the ART+As_2_O_3_ group throughout the whole study period. (**G–J**) Viral load (plasma SIV RNA) levels of the four macaques in the ART +saline group during the whole study period. The gray areas represent the period of ART treatment, while the pink areas represent the period of As_2_O_3_ treatment, and the blue areas represent the period of saline treatment. (**K**) The survival curves of the two groups were analyzed by log-rank (Mantel-Cox) tests. (**L and M**) Changes in CD4^+^ T cell and CD8^+^ T cell counts in the ART+As_2_O_3_ and ART +saline groups before and after treatment. *P < 0.05, **P < 0.01, ***P < 0.001.

To explore the advantages of launching As_2_O_3_ in HIV/SIV acute infection, we investigated the effects of ART+As_2_O_3_ combination therapy in acutely SIVmac239-infected rhesus macaques. To establish the acutely SIV-infected macaque model, nine rhesus macaques were inoculated intravenously with 5,000 50% tissue culture infectious dose (TCID_50_) of SIVmac239. Macaques were confirmed to be positive for SIV infection by two independent tests for plasma viral load. All macaques were successfully infected and had a detectable SIV load beginning on day 4 after the challenge (Fig. S3A). All acutely SIV-infected macaques were assigned into two groups: the ART+As_2_O_3_ group (*n* = 5) and the ART +saline group (*n* = 4) (Fig. 5A; ). On day 7 after challenge (i.e., day 3 after infection was confirmed), all macaques were given an ART regimen consisting of daily subcutaneous injection of 9-[2-(R)-(phosphonomethoxy) propyl] adenine (PMPA, 30 mg/kg) and emtricitabine (FTC, 20 mg/kg) for 17 d. On day 13 after the challenge (i.e., day nine after infection was confirmed), As_2_O_3_ (Arsenic Trioxide for Injection, H20080664, SL PHARM, 0.16 mg/kg) was given intravenously twice daily for 8 days and once daily for the last 3 days.

The dynamics of plasma viral load in acutely SIV-infected macaques were tested by real-time quantitative PCR as reported previously (29). In our acutely SIV-infected macaque model, the plasma viral load dropped rapidly during ART, but the viral load fluctuated and rebounded after ART discontinuation. Consistent with our previous observation in chronically SIV-infected macaques, ART+As_2_O_3_ treatment delayed viral rebound after ART discontinuation compared with ART +saline treatment (Fig. 5B through J). A total of three out of five SIV-infected macaques (RM6, RM7, RM10) treated with ART+As_2_O_3_ achieved a relatively stable period of viremia remission after 53 days of ART discontinuation (i.e., 76 days post infection) (Fig. 5B through F). The average time of the first viral load rebound after ART interruption in three macaques (RM6, RM7, RM10) was longer than that of the ART +saline group (Fig. 5B, C and F). In particular, the viral load in these three macaques did not reach a stable undulating state (setpoint) at the end point (Fig. 5B, C and F; Fig. S3B). In addition, the area under curve (AUC) value representing the area under the plasma viral load curve was calculated. We found that the AUC values of the three macaques (RM6, RM7, RM10) in the ART+As_2_O_3_ group were lower than those in the ART +saline group, indicating that these macaques had better control of disease progression (Fig. S3C).

To evaluate the latency reversal by As_2_O_3_, we defined it as on-ART plasma viremia increasing from less than 100 copies/mL, which is the limit of our detection, to more than 100 copies/mL after at least a 7 days viremia remission. Two of five macaques (RM6 and RM10) showed latency reversal during the period of ART+As_2_O_3_ treatment. These two macaques also had a slow progression of viremia (Fig. 5B and F). In contrast, a short period of ART +saline treatment was not sufficiently effective to control the progression of SIVmac239 infection. Two macaques (RM12 and RM13) had a dramatic reduction in CD4^+^ T cells and died on day 22 and day 44 after infection, respectively (50% survival rate) (Fig. 5H, I and K). The viral load of RM14 decreased during ART treatment and showed a typical increase after ART interruption (Fig. 5J), while that of RM11 remained at 10^4^ copies/mL after ART (Fig. 5G). Overall, these results indicated that ART+As_2_O_3_ therapy can effectively delay the progression of viremia and increase the survival rate in acutely SIVmac239-infected macaques after ART interruption.

T cell count, especially the quantity of CD4^+^ T cells, plays an independent role in the evaluation of the disease progression of AIDS patients and can reflect the health conditions of the immune system (30). The results showed that the number of CD4^+^ T (Fig. 5L) and CD8^+^ T (Fig. 5M) cells in ART+As_2_O_3_ treated rhesus macaques increased significantly throughout the study period, and the CD4/CD8 ratio of these macaques was greater than 0.8 after treatment (Fig. S3D). In contrast, the number of T cells in the ART + saline group also showed an upward trend, but no significant difference was observed (Fig. 5L and M). One of the macaques (RM14) in the ART + saline group had a CD4/CD8 ratio of only 0.46 at the last monitoring point, which decreased 64% compared with 1.27 before SIV infection (Fig. S3D). Notably, the CD4^+^ T, CD8^+^ T cell counts, and the CD4/CD8 ratios increased significantly in three out of five rhesus macaques (RM6, RM7, RM10) that had well-controlled viremia in the ART+As_2_O_3_ group (Fig. S3F through H).

### ART+As_2_O_3_ therapy improved SIV-specific immune responses without excessive immune activation in acutely SIVmac239-infected macaques

**Fig 6:**
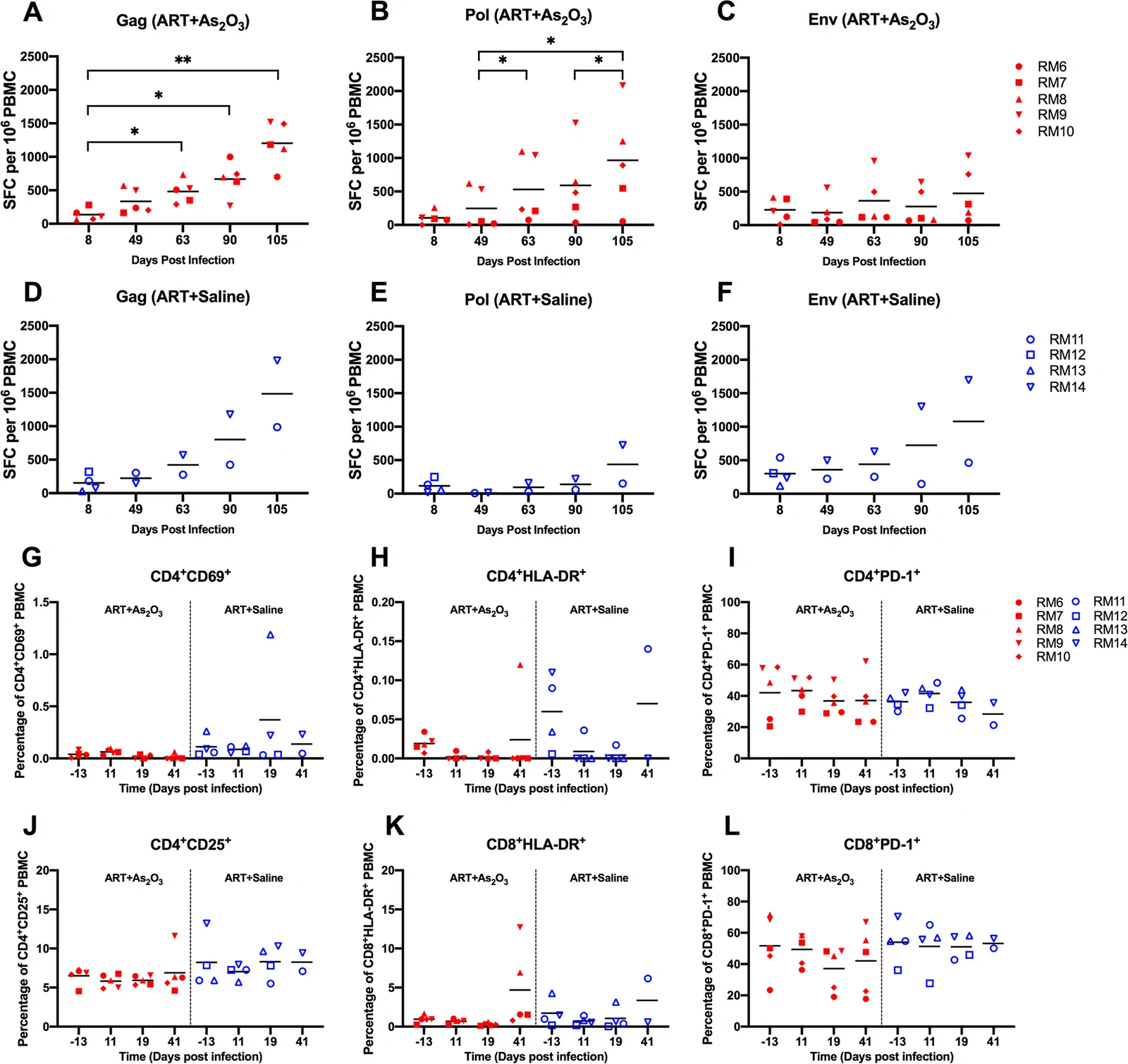
ART + As_2_O_3_ therapy improved SIV-specific immune responses without excessive immune activation in acutely SIVmac239-infected macaques. The specific IFN-γ-secreted spot-forming cells (2) per million PBMCs at different time points in two groups were measured by ELISpot assay. (**A–C**) SIVmac239 Gag-, Pol-, and Env-specific total IFN-γ-secreted spot-forming cells (SFCs) per million PBMCs of the ART+As_2_O_3_ group at equivalent time points. (**D–F**) SIVmac239 Gag-, Pol-, and Env-specific total IFN-γ-secreted SFCs per million PBMCs of the ART + saline group at equivalent time points. All data are presented as the grand mean. (**G–L**) The expression level of T cell activation markers in all macaques was detected through flow cytometry. (**G**) Percentage of CD4^+^CD69^+^ cells. (**H**) Percentage of CD4^+^HLA-DR^+^ cells. (**I**) Percentage of CD4^+^PD-1^+^ cells. (**J**) Percentage of CD4^+^CD25^+^ cells. (**K**) Percentage of CD8^+^HLA-DR^+^ cells. (**L**) Percentage of CD8^+^PD-1^+^ cells. All data are presented as the grand mean. *P < 0.05, **P < 0.01.

To assess the effect on the SIV-specific T cell immune response by ART+As_2_O_3_ therapy, an IFN-γ-mediated enzyme-linked immunosorbent spot (ELISpot) assay was performed throughout the study period. We found that ART+As_2_O_3_ therapy could significantly improve the SIVmac239 Gag- and Pol-specific immune response after ART withdrawal (Fig. 6A and B), while the SFCs in the ART +saline group also showed an upward trend after ART interruption (Fig. 6D through F). In addition, Env-specific SFCs also showed an upward trend after ART+As_2_O_3_ treatment, but there was no significant change (Fig. 6C).

Persistent and excessive T cell activation is one of the essential factors that leads to T cell exhaustion in HIV patients (31). To evaluate the activation status of T cells, we measured the expression of various activation markers on the surface of CD4^+^ and CD8^+^ T cells through flow cytometry. The results showed that the expression of CD69, HLA-DR, PD-1, and CD25 on CD4^+^ T cells did not change significantly during ART+As_2_O_3_ treatment, and there was no significant difference between the two groups (Fig. 6G through J). Notably, there was a trend toward a reduced expression of CD69 on the surface of CD4^+^ T cells in the ART+As_2_O_3_ group after treatment interruption compared with the ART + saline group (Fig. 6G). Meanwhile, the expression of HLA-DR and PD-1 on the surface of CD8^+^ T cells was similar in the two groups of macaques (Fig. 6K and L).

Taken together, ART+As_2_O_3_ treatment improved T cell function more significantly with a lower level of T cell activation in acutely SIVmac239-infected macaques.

## DISCUSSION

As_2_O_3_ was notorious for a long time due to its toxicity and was once called “*Poudre De Succession*” by the French, which means a poison to scheme for royal inheritance (https://www.etf.com). Even so, its therapeutic potential was uncovered both in ancient China and Western countries (32). Most notably, As_2_O_3_ was approved as a new therapeutic reagent for treating acute promyelocytic leukemia (APL) by the National Medical Products Administration (NMPA) of China in 1999 (33). In addition, synergistic targeted therapy with all-trans retinoic acid (ATRA)/As_2_O_3_ was recommended as the first choice for APL treatment by the USA National Comprehensive Cancer Network (NCCN) in 2014 (34, 35). It has also been approved for the clinical treatment of hepatocellular carcinoma (36). In our previous study, we proved the potential of HIV/SIV latency reversal by As_2_O_3_ and its therapeutic effects in chronically SIVmac239-infected rhesus macaques when combined with ART (12). In the present study, to further clarify the effects and mechanisms of As_2_O_3_ in HIV/SIV treatment, we treated chronically SIVmac239-infected rhesus macaques with As_2_O_3_ alone. The results showed that As_2_O_3_ reactivated the viral latency *in vivo*, which was reflected by the blip of viral load and the elevated level of SIV gag total DNA and cell-associated SIV RNA. SIV-specific immune responses toward structural genes were enhanced after As_2_O_3_ injection. The comparisons of the results between our previous study and the present study are listed in Table S2.

One of the concerns about As_2_O_3_ application in AIDS treatment is the overall T cell loss caused by such a proapoptosis agent (37). As_2_O_3_ was reported to induce cell apoptosis by collapsing the mitochondrial membrane potential in acute promyelocytic leukemia (38), enhance the TRAIL-induced apoptosis of glioma cells through the upregulation of DR5 (39), and induce apoptosis by upregulating p53 and activating caspase-3 in human gastric cancer cells (40). Herein, we observed its apoptosis-inducing effect *in vivo*. The number of T cells showed a decreasing trend during the injection period; however, it recovered to the initial level or even a higher level once the treatment was stopped. Actually, the transient reduction in CD4^+^ T cells might shrink the viral reservoir, as we first found that As_2_O_3_ treatment tended to induce the apoptosis of latent HIV/SIV-infected cells. When compared with Jurkat cells, J-Lat10.6 cells witnessed a higher level of apoptosis upon As_2_O_3_ stimulation. The apoptotic level of CD4^+^ T cells from macaques with severe viremia was higher than that from healthy donors after As_2_O_3_ treatment. We demonstrated that As_2_O_3_ not only reactivates the viral reservoir but also induces the apoptosis of HIV/SIV-infected cells, which broadens its application in the “shock-and-kill” strategy.

According to the results from RNA-seq analyses, genes related to viral entry and infection were downregulated, which indicated that the infection rate of bystanders could be decreased. On the other hand, the upregulation of host restriction factors can also protect healthy cells from infection. It was reported that tetherin was capable of inhibiting the release of HIV-1 by directly tethering virions to cells (25). Therefore, we speculated that although the latent reservoir was reactivated by As_2_O_3_ stimulation, the budding virions might not be released due to the upregulated expression of tetherin. This hypothesis is worthy of investigation in our further study. Downregulation of the viral receptor CCR5 on CD4^+^ T cells was also observed here. The mechanism behind this phenomenon is of great significance, as CCR5 is considered to be a key factor in achieving an HIV functional cure (41, 42). It was reported that gag stimulation could downregulate the expression of CCR5 on CD4^+^ T cells through β-chemokine production (22). Although intrinsic explanations are still absent, they help us to link the increased SIV transcripts caused by As_2_O_3_ stimulation with the lower level of CCR5. This study also revealed that reactivation of the SIV reservoir upon As_2_O_3_ stimulation might be associated with the downregulation of PML. It was reported that As_2_O_3_ controlled the fate of the promyelocytic leukemia RA receptor α (PML-RARα) oncoprotein by directly binding PML (43). Other studies have noted that the degradation of PML and nuclear body disruption resulted in strong activation of HIV transcription (20). PML was also reported to sequester cyclin T1, which is a subunit of the cellular transcription elongation factor p-TEFb (44, 45). It seems that As_2_O_3_ stimulation can impact the nuclear topology to affect viral transcription. We also found that As_2_O_3_-induced reactivation of the viral reservoir may be caused by the induction of NF-κb, which was previously reported by another study (13).

Some studies have revealed the benefits of initiating ART in the acute HIV infectious phase. It was reported that the HIV reservoir reached a maximal value before seroconversion in gut-associated lymphoid tissues and lymph nodes. Initiation of ART before the Fiebig stage III cleared the majority of infected cells in blood and tissues (46). The HIV DNA set point was also reduced dramatically by early ART (47). Another large cohort study that included 321 patients with acute or early HIV infection demonstrated that early ART enhanced CD4^+^ T-cell recovery (48). The function of mucosal Th17 cells was preserved, and HIV-related immune activation was reversed by early ART (49). Despite the positive therapeutic effects of early ART, it is not enough to eradicate the HIV reservoir. It was reported that the viral load rebounded in a median of 26 days after ART interruption, even when initiated as early as the Fiebig stage II (HIV RNA^+^, p24^-^, IgM^-^) (50). Therefore, it is more promising to achieve an HIV functional cure by combining ART with other therapeutic agents in acute HIV infection.

We explored the effects of ART+As_2_O_3_ treatment in acutely SIVmac239-infected rhesus macaques for the first time. Notably, three of five enrolled macaques in the ART+As_2_O_3_ group exhibited better control of viremia, as well as increased T cell numbers throughout the study period. We observed that the specific immune responses of the two groups differed based on the different antigen peptides, and further studies are needed to uncover the underlying mechanisms.

One limitation of this study is the relatively small number of SIVmac239-infected macaques. Moreover, after ART discontinuation in acutely SIVmac239-infected macaques, there were only two survived control animals in ART +saline group due to the death of RM12 and RM13, which made it difficult to draw significant conclusions to compare with therapeutic effect in ART+As_2_O_3_ group. Nevertheless, we analyzed the viral load of each macaque longitudinally to comprehensively understand the effects of As_2_O_3_ treatment, and we also conducted experiments to explore the therapeutic effects of As_2_O_3_ treatment alone in chronically SIVmac239-infected macaques. In the future, more animals are needed to further confirm our conclusion, and the exact mechanism behind the therapeutic effects of As_2_O_3_ should be further clarified.

In conclusion, our study explored the therapeutic potential of ART+As_2_O_3_ treatment in both chronically (12) and acutely SIVmac239-infected rhesus macaques, as well as the effects of As_2_O_3_ when applied alone. These studies may provide guidance for our ongoing phase I clinical trial toward people living with HIV at the Guangzhou Eighth People’s Hospital, Guangzhou Medical University in China (https://clinicaltrials.gov/ct2/show/NCT03980665). More importantly, this study provides insights into the development of novel “shock-and-kill” strategies to achieve HIV functional cure.

## MATERIALS AND METHODS

### Virus, SIVmac239 peptides, and arsenic trioxide

The SIVmac239 stock used in this study was the same as we previously reported (51). SIVmac239 peptides were general gifts from the NIH AIDS Research and Reference Reagent Program, which is now known as the NIH HIV Reagent Program (www.hivreagentprogram.org). The peptide pools included the entire sequence of SIVmac239 Gag, Pol, and Env proteins. The length of these peptides was 15 amino acids with 11 overlapping residues. All peptides were dissolved in dimethyl sulfoxide (Sigma, Cat. No. D2650) to a concentration of 0.4 mg/mL as stock solutions and stored at −80°C. The working concentration of peptides was 0.002 mg/mL. Arsenic trioxide for injection (H20080664) was obtained from SL PHARM (002038. SZ, Beijing, China). This product was approved by the National Medical Products Administration, formerly the China Food and Drug Administration, in 2013 for the treatment of acute promyelocytic leukemia (APL). The concentration adopted in this study is the same as the suggested dosage for APL treatment, which is 0.16 mg/kg.

### Animals and drug administration

In this study, Chinese rhesus macaques (*Macaca mulatta*) were used as the animal model. nine chronically SIVmac239-infected macaques with viral loads less than 4 log10 were randomly assigned to two groups, except for one of them with a significantly lower viral load (≤2 log10), which was assigned to the As_2_O_3_-only group. Five macaques were treated with As_2_O_3_ (Arsenic Trioxide for Injection, H20080664, SL PHARM, 0.16 mg/kg) alone by intravenous drip daily for 12 days, while four macaques were untreated as controls (Fig. 1A). For the acutely SIV-infected animal experiment included in this study, nine macaques were challenged by SIVmac239 (5,000 TCID_50_) intravenously on day 0. According to the age and weight of the macaques, five of them were assigned to the ART+As_2_O_3_ group, while four macaques were assigned to the ART +saline group. As_2_O_3_ (0.16 mg/kg) treatment started on day 13 and stopped on day 23. It was given intravenously twice a day for 8 days and once daily for the last 3 days. ART regimen consisted of daily subcutaneous injection of 9-[2-(R)-(phosphonomethoxy) propyl] adenine (PMPA, 30 mg/kg) and emtricitabine (FTC, 20 mg/kg), and was initiated for all of the macaques on day seven and was withdrawn at day 23 (Fig. 5A).

### Assays for evaluating cellular immune responses

We evaluated SIVmac239-specific immune responses through the IFN-γ-mediated ELISpot assay as we previously reported (52). Briefly, a 96-well PVDF plate (Merck Millipore, Cat. No. MSBVS1210) was coated with monoclonal coating antibodies (U-CyTech, Cat. No. CT605-10) overnight at 4°C. PBMCs were isolated through density gradient centrifugation by Lymphoprep (Axis-Shield, Cat. No. 1114547). The plate was washed three times with DPBS (BasalMedia, Cat. No. B210KJ) and 200 µL per well of R10 medium (RPMI 1640, 10% fetal bovine serum, 1% penicillin‒streptomycin, 1 mM sodium pyruvate, 2 mM L-glutamine, 0.05 mM 2-mercaptoethanol, and 10 mM HEPES) were added to block the plate for 2 h. PBMCs were diluted with R10 medium, and 300,000 PBMCs were added to each well. The SIVmac239 peptide stocks were diluted 20 times by R10, and 10 µL of each peptide was added to the corresponding well. After incubation for 24 h, the plate was washed, and biotinylated monoclonal detection antibodies (U-CyTech, Cat. No. CT605-10) were added. The next morning, streptavidin-AKP (BD PharMingen, Cat. No. 554065) was added and incubated for 2 h at 37°C. The 1-STEP NBT/BCIP (Thermo Scientific, Cat. No. 34042) was then added and incubated for 10 min at 37°C. The plate was washed and dried before being read by an enzyme-linked immunospot imager (Bioreader6000, BIOSYS, Germany).

### CD4^+^ T cell sorting by magnetic-activated cell sorting

We used CD4 MicroBeads for nonhuman primates (Miltenyi Biotec, Cat. No. 130–091-102) to isolate CD4^+^ T cells from PBMC samples of rhesus macaques. Two kinds of DPBS buffer (5% FBS, 2 mM EDTA, and 2% FBS, 2 mM EDTA) were prepared in advance. PBMCs were magnetically labeled with CD4 microbeads. The usage of buffer and microbeads depended on the total number of PBMCs collected. Then, the mixture of cells and beads was incubated for 15 min at 4°C. The cells were washed using 0.5% FBS DPBS buffer and centrifuged at 300× g for 10 min. Resuspension of the cells with 2% FBS DPBS buffer increased the survival rate. We used MS magnetic-activated cell sorting (MACS) columns to proceed with the magnetic separation. Columns were placed in the magnetic field of a MACS Separator and rinsed with 3 mL of 2% FBS DPBS buffer. The cell suspensions were then applied to the columns. The columns were washed three times with the same volume of buffer. Then, the columns were removed from the separator and placed in a collection tube. CD4^+^ T cells were collected after rinsing the columns with 9 mL of 2% FBS DPBS buffer.

### Detection of apoptotic cells

We evaluated the percentage of apoptotic cells through flow cytometry. For the detection of early stage apoptotic CD4^+^ T cells, we measured the expression of membrane phospholipid phosphatidylserine (PS) through Annexin V conjugated to FITC fluorochrome (BD Pharmingen, Cat. No. 556547). The vital dye propidium iodide (PI) was also added to help identify early apoptotic cells. PI-positive and Annexin V-positive cells were regarded as advanced-stage apoptotic cells in this study. As the J-Lat10.6 cell line contains green fluorescent proteins, which occupy the same fluorescent light channel as FITC fluorochrome, we adopted another PE Annexin V Apoptosis Detection Kit I (BD Pharmingen, Cat. No. 559763) to evaluate the apoptosis of Jurkat and J-Lat10.6 cell lines. The vital dye 7-amino-actinomycin (7-AAD) was used in this kit to help distinguish different subtypes of cells. Early apoptotic cells were defined as Annexin V-positive and 7-AAD-negative, while advanced apoptotic cells were defined as Annexin V/7-AAD-double-positive. All data were collected using an LSRFortessa (BD Biosciences) instrument and were analyzed using FlowJo software (Tree Star, Inc).

### SIV RNA and DNA copy assays

The viral load, represented by the SIV RNA copies in plasma, was detected as we previously described (53). RNA was extracted by a QIAamp viral RNA mini kit (Qiagen, Cat. No. 52906) and was quantified using the QuantiTect SYBR Green RT‒PCR Kit (Qiagen, Cat. No. 204245). For cell-associated RNA detection, RNA from PBMCs was separated using the EastepTM Super Total RNA Extraction Kit (Promega, Cat. No. LS1040) and then reverse transcribed using GoScript Reverse Transcription Mix, Random Primers (Promega, Cat. No. A2800). The first round of PCR was performed using Premix Taq (Takara, Cat. No. R004A). The following second real-time fluorescent quantitative PCR was performed using ChamQ SYBR qPCR Master Mix (Vazyme, Cat. No. Q311-02). For SIV total gag DNA detection, genomic DNA was extracted from PBMCs by a Rapid Genomic DNA Kit (Biomed, Cat. No. DL110-01) and was quantified using ChamQ SYBR qPCR Master Mix (Vazyme, Cat. No. Q311-02). All final data were collected and analyzed using a CFX96 Real-Time PCR system (Bio-Rad).

### T cell counts and phenotypic characteristic assays

Blood samples from rhesus macaques were all collected following standard protocols. BD Trucount Absolute Count Tubes (BD, Cat. No. 340334) were used to quantify the T cells in 100 µL of blood. Antibodies that label different kinds of T cells were adopted in this study. Information on antibodies is listed in Table S3. FACS Lysing solution (BD Cat. No. 349202) was diluted 10 times before being added, and the suspensions were incubated at 4°C for 10 min. All data were collected through BD Accuri C6 Plus. PBMCs were collected to test the phenotypic characteristics of T cells, and the antibodies used are all listed in Table S3. All data were collected by an LSRFortessa (BD Biosciences) instrument and were analyzed using FlowJo software (Tree Star, Inc).

### RNA sequencing and bioinformatic analyses

The transcriptomics analysis by RNA-seq was similar to that we reported previously (53). Briefly, 1 µg of RNA from each sample was used as input material for this study, and the RNA integrity was evaluated using the RNA Nano 6,000 Assay Kit of the Bioanalyzer 2,100 system (Agilent Technologies, CA, USA). The cDNA library was constructed and accessed before sequencing by an Illumina NovaSeq platform. Reads containing adapter, poly-N, and low-quality reads from raw data were removed before further analysis. Clean reads were then mapped and aligned to the *Macaca mulatta* genome using Hisat2 v2.0.5. We adopted StringTie (v1.3.3b) to assemble the mapped reads. The read number mapped to each gene was counted by featureCounts v1.5.0-p3. The gene expression level was represented by the expected number of fragments per kilobase of transcript sequence per million base pairs sequenced (FPKM), which was calculated according to the gene length and read number mapped to this gene. As we had four biological replicates in each group, we used the DESeq2 R package (1.20.0) to perform the differential expression analysis. Genes with a *P*-value < 0.05 were considered differentially expressed. In addition, we performed gene set enrichment analysis (GSEA) using data from the KEGG database.

### Statistical analysis

GraphPad Prism 8 software (GraphPad Software Inc., La Jolla, CA, USA) was used for analysis. Comparisons between groups were performed using two-tailed unpaired *t*-tests. Comparisons between two time points in the same group were performed using two-tailed paired *t*-tests. For multiple comparison analyses, analysis of variance (ANOVA) followed by post-hoc tests were performed. The differences were considered significant when *P*-values < 0.05.

## Data Availability

Data supporting the findings of this study are available in the supplemental material online Table S4.

## SUPPLEMENTAL MATERIAL

Fig.S1 to S3, Table S1 to S3 spectrum.00525-23-s0001.docx Supplemental materials.

Table S4 spectrum.00525-23-s0002.csv

ASM does not own the copyrights to Supplemental Material that may be linked to, or accessed through, an article. The authors have granted ASM a non-exclusive, world-wide license to publish the Supplemental Material files. Please contact the corresponding author directly for reuse.
