# Supplementary material for: Arsenic trioxide-induced apoptosis contributes to suppression of viral reservoir in SIV-infected rhesus macaques
Source: Microbiol Spectr. 2023 Sep 11;11(5):e00525-23. doi: 10.1128/spectrum.00525-23 (PMC10581169; doi:10.1128/spectrum.00525-23)
Supplement: Fig.S1 to S3, Table S1 to S3 — Supplemental materials. [file spectrum.00525-23-s0001.docx]

**SUPPLEMENTARY MATERIALS**


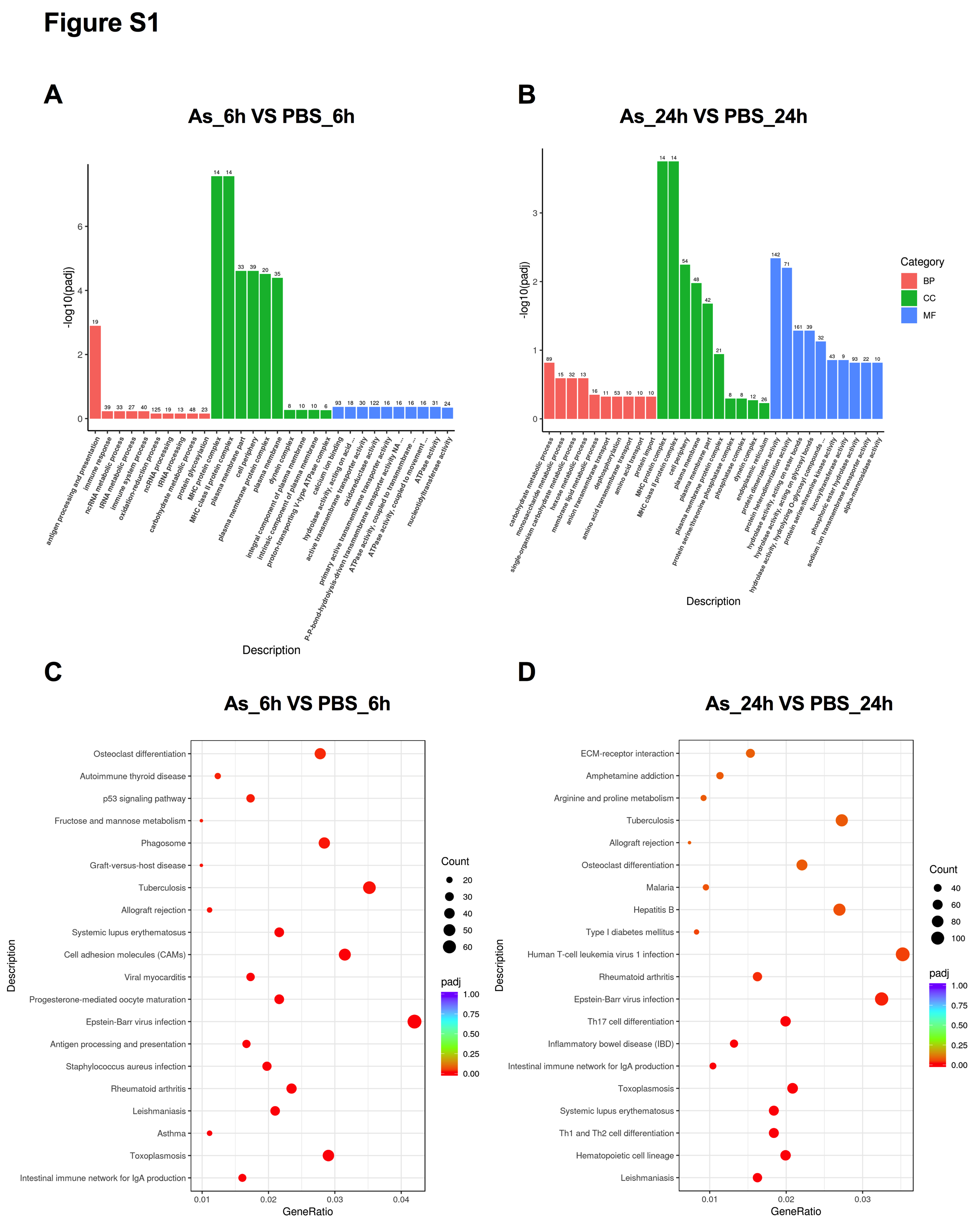


**FIG S1 GO and KEGG enrichment analysis of differentially expressed genes.**

ClusterProfiler R packages were used to test the statistical enrichment of differentially expressed genes (DEGs) in the GO (Gene Ontology) and KEGG (Kyoto Encyclopedia of Genes and Genomes) databases. The top 30 pathways in GO enrichment analysis toward DEGs after As_2_O_3_ treatment and PBS treatment for 6 hours and 24 hours are shown separately in **(A, B)**. BP represents biological process, CC represents cellular component and MF represents molecular function. **(C, D)** Top 20 enriched pathways in the KEGG database after As_2_O_3_ treatment and PBS treatment for 6 hours and 24 hours. The abscissa in the figure is the ratio of the number of DEGs annotated to the KEGG pathway to the total number of DEGs, while the ordinate is the KEGG pathway. The size of the dots represents the number of genes annotated in the KEGG pathway, and colors from red to purple represent the significance of enrichment from large to small.

**
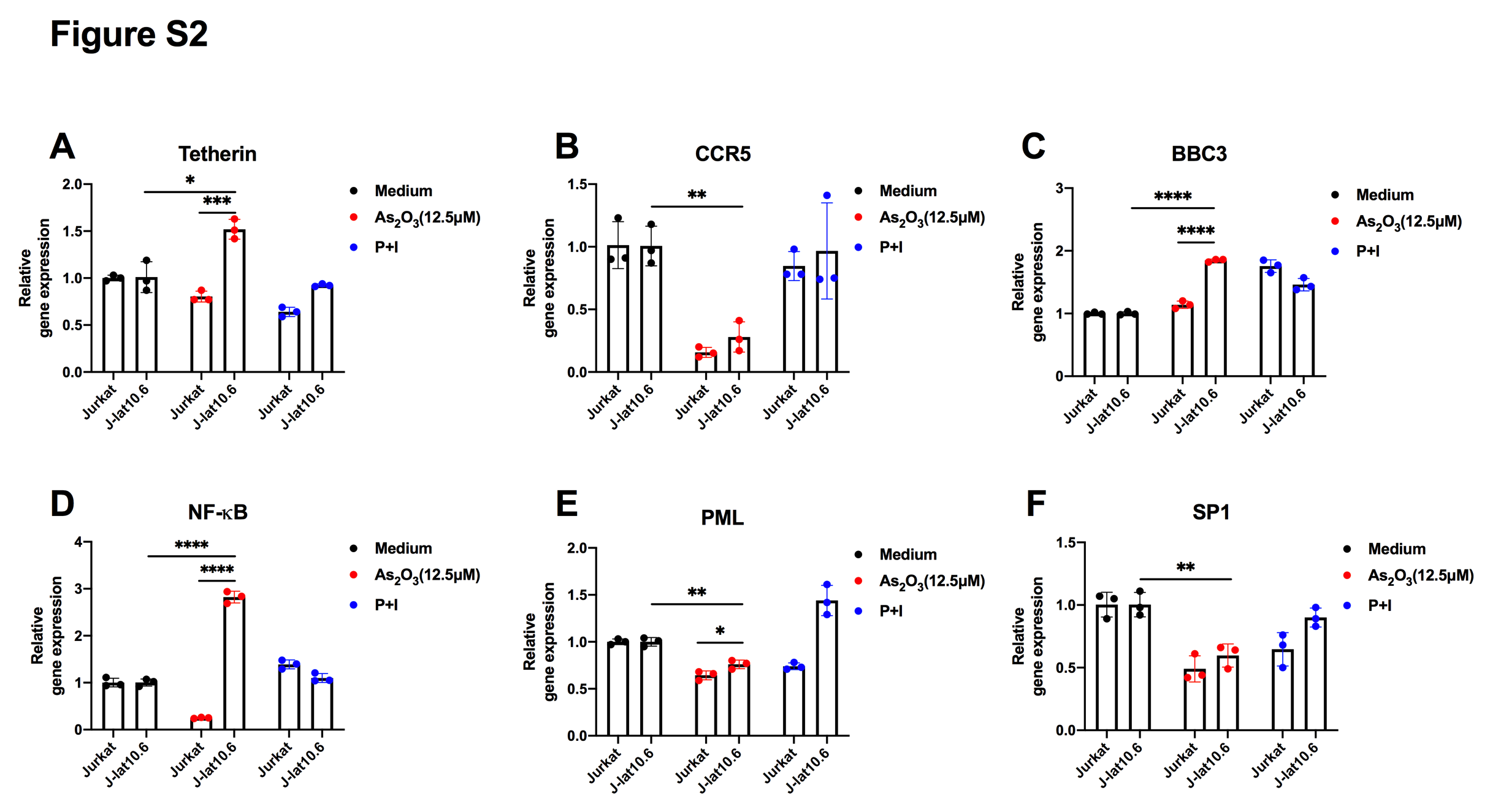
**

**FIG S2 As_2_O_3_ treatment upregulated the expression level of key genes in J-Lat10.6.**

Jurkat and Jurkat-Lat HIV-1 full-length clone A10.6 cell lines were pretreated with medium, As_2_O_3_ (12.5 μM) and P+I (phorbol myristate acetate 40 ng/ml+ionomycin 1000 ng/ml) for 24 hours. Then, the cells were collected to extract the RNA, and quantitative PCR was performed after reverse transcription. **(A)** The relative expression level of host restriction factor Tetherin after drug treatment. **(B)** The relative expression level of viral receptor CCR5. **(C)** The relative expression level of proapoptotic gene BBC3. **(D-F)** The expression level of genes (NF-κb, PML and SP1) related to the transcription of HIV/SIV reservoir. Each experiment was performed once with three replicates. The black dots represent the medium-treated samples, the red dots represent the As_2_O_3_-treated samples, and the blue dots represent the P+I-treated samples. Each experiment was performed in triplicate. All data are presented as the mean with SD (*p<0.05, **p<0.01, ***p<0.001, ****p<0.0001).

**
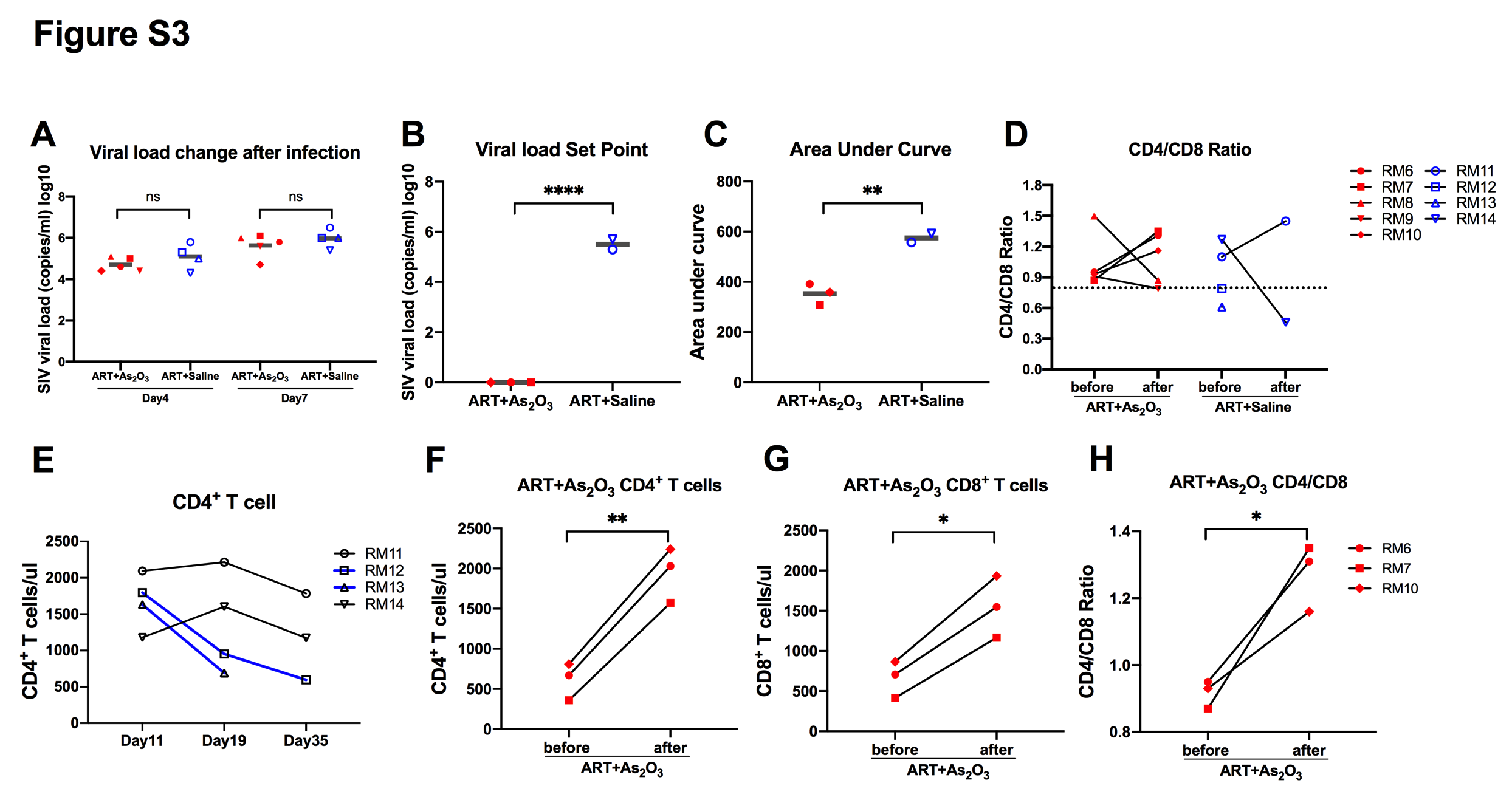
**

**FIG S3 ART+As_2_O_3_ therapy delayed the progression of viremia and improved the CD4/CD8 ratio in three acutely SIVmac239-infected rhesus macaques after ART interruption.**

**(A)** All macaques were infected successfully, and the viral load of all macaques could be detected from day 4. The initial viral load of macaques in two groups at day 7 was not significantly different. The data are presented as the grand mean. **(B)** Comparison of the viral load set point between the three macaques with delayed progression of viremia in the ART+As_2_O_3_ group and the two surviving macaques in the ART+Saline group. **(C)** Comparison of the area under curve value of viral load between the three macaques with delayed progression of viremia in the ART+As_2_O_3_ group and the two surviving macaques in the ART+Saline group. **(D)** Changes in the CD4/CD8 ratio of the ART+As_2_O_3_ and ART+Saline groups before and after treatment. A CD4/CD8 ratio threshold of >0.8 was set to evaluate the health status of all macaques. **(E)** The dynamics of CD4^+^ T cell counts of the macaques in the ART+Saline group from day 11 to day 35. **(F-H)** Changes in CD4^+^ T cell and CD8^+^ T cell counts and CD4/CD8 ratio of the three macaques (RM6, RM7, and RM10) with delayed progression of viremia in the ART+As_2_O_3_ groups before and after treatment. *p<0.05, **p<0.01, ***p<0.001, ****p<0.0001.

**Table S1. Information of rhesus macaques**

| **groups** | **ID** | **Gender** | **Weight (kg)** | **Age (year)** |
| --- | --- | --- | --- | --- |
|  |  |  |  |  |
| **As_2_O_3_-only** | RM1 | male | 8.12 | 15 |
|  | RM2 | female | 7.8 | 7 |
|  | RM3 | male | 10.8 | 16 |
|  | RM4 | male | 9.4 | 12 |
|  | RM5 | male | 10.0 | 8 |
| **ART+As_2_O_3_** | RM6 | male | 12.7 | 6 |
|  | RM7 | male | 10.5 | 6 |
|  | RM8 | male | 13.48 | 6 |
|  | RM9 | male | 8.75 | 13 |
|  | RM10 | male | 9.46 | 6 |
| **ART+Saline** | RM11 | male | 9.76 | 6 |
|  | RM12 | female | 7.14 | 16 |
|  | RM13 | male | 16.98 | 6 |
|  | RM14 | male | 10.86 | 6 |

**Table S2. Comparison between chronically and acutely SIV-infected macaques after ART+As_2_O_3_ treatment**

| **Test index after ART+As_2_O_3_**  **treatment** | **Chronically**  **SIVmac239-infected macaques** | **Acutely**  **SIVmac239-infected macaques** |
| --- | --- | --- |
|  |  |  |
| Delay of disease progression | 2/4 | 3/5 |
| CD4^+^ T cell count | Increased | Increased |
| Antigen-specific IFN-γ immune response | Elevated | Elevated |
| Expression of activation marker on T cells (CD69 etc.) | No significant change | No significant change |

**Table S3. Information of antibodies used for flow cytometry in this study**

| **Objective** | **Product Name** | **Clone** | **Brand** |
| --- | --- | --- | --- |
|  |  |  |  |
| **T cell count** | FITC Mouse Anti-Human CD4 | L200 | BD Pharmingen™ |
|  | PercP Mouse Anti-Human CD8 | SK1 | BD Pharmingen™ |
|  | APC Mouse Anti-Human CD3 | SP34-2 | BD Pharmingen™ |
|  | PE Mouse Anti-NHP CD45 | D058-1283 | BD Pharmingen™ |
| **Phenotypic analysis of T cells** | Pacific Blue Mouse Anti-Human CD3 | SP34-2 | BD Pharmingen™ |
|  | PE-CF594 Mouse Anti-Human CD4 | L200 | BD Pharmingen™ |
|  | FITC Mouse Anti-Human CD4 | L200 | BD Pharmingen™ |
|  | APC-cy7 Mouse Anti-Human CD8 | RPA-T8 | BD Pharmingen™ |
|  | APC Mouse Anti-Human CD8 | RPA-T8 | BD Pharmingen™ |
|  | APC Mouse Anti-Hu/NHP CD25 | CD25-4E3 | eBioscience |
|  | PE-eF610 Mouse Anti-Human CD279 | eBioJ105 | eBioscience |
|  | PE Mouse Anti-Human HLA-DR | G46-6 | BD Pharmingen™ |
|  | FITC Mouse Anti-Human CD69 | FN50 | BD Pharmingen™ |
